# Quality of life and depression in Wilson’s disease: a large prospective cross-sectional study

**DOI:** 10.1186/s13023-023-02777-4

**Published:** 2023-06-29

**Authors:** Kevin Chevalier, Djamila Rahli, Louise de Veyrac, Jessica Guillaume, Michaël Alexandre Obadia, Aurélia Poujois

**Affiliations:** 1 Department of Neurology, Adolphe de Rothschild Foundation Hospital, Paris, France; 2 National Reference Center for Wilson’s Disease and Other Copper-Related Rare Diseases, Adolphe de Rothschild Foundation Hospital, 29 Rue Manin, 75019 Paris, France; 3 Clinical Research Department, Adolphe de Rothschild Foundation Hospital, Paris, France

**Keywords:** Wilson’s disease, Quality of live, Depression

## Abstract

**Background:**

Wilson's disease (WD) is an autosomal recessive genetic disorder due to a mutation of the *ATP7B* gene, resulting in impaired hepatic copper excretion and accumulation in various tissues. Lifelong decoppering treatments are the keystone of the treatment. These treatments can prevent, stabilize, or reverse the symptoms making WD a chronic disease. Quality of life (QoL) is one of the best outcome measures of any therapeutic intervention in chronic diseases but has not been evaluated in large cohorts of WD patients.

**Method:**

To better evaluate the QoL in WD and the correlation with different clinical or demographic factors we have performed a prospective cross-sectional study.

**Results:**

Two hundred fifty-seven patients (53.3% men, mean age of 39.3 years and median disease duration of 18.8 years) were included between 1st January 2021 and 31st December 2021. Hepatoneurological form of the disease and depression were significantly correlated with low QoL (*p* < 0.001 for both). However, the patients' quality of life was similar to that of the general population, and only 29 patients (11.3%) had moderate to severe depression.

**Conclusions:**

Neurological patients should be closely monitored to prevent and treat symptoms of depression that impact their quality of life.

**Supplementary Information:**

The online version contains supplementary material available at 10.1186/s13023-023-02777-4.

## Introduction

Wilson's disease (WD) is a rare genetic condition due to a recessive mutation of the *ATP7B* gene. The disease consists of a continuous copper accumulation in many tissues. This condition, mainly characterized by hepatic, ophthalmological and neurological features could lead to severe disability or even death if the diagnosis is delayed or the treatment poorly taken. Lifelong treatments including chelators and zinc salts are the keystone of the treatment and can prevent, stabilize, or reverse the copper accumulation and the symptoms of the disease making WD a chronic disease. Quality of life (QoL) is one of the best outcome measures of any therapeutic intervention in chronic diseases but has not been evaluated in large cohorts of WD patient. To better evaluate the QoL in WD and the correlation with different clinical or demographic factors we have performed a monocentric prospective cross-sectional study.

## Materials and methods

WD patients from the Wilson's Disease Reference Center in Rothschild Foundation Hospital (Paris, France) aged > 18 years old, have been evaluated between 01/01/2021 and 31/12/2021. After giving their consent, the QoL of patients have been evaluated thanks to the EQ-5D-5L questionary. EQ-5D-5L is a standardized measure of health status developed by the EuroQoL Group to provide a simple, generic measure of health for clinical and economic appraisal [1]. The EQ-5D-5L version (Additional file 1: EQ-5D-5L questionary) is currently the recommended tool to measure the QoL by the French National Authority for Health (Haute Autorité de Santé). It consists in a self-questionary evaluating five dimensions (mobility, self-care, usual activities, pain/discomfort, and anxiety/depression), each with five response levels from 1 (no problems) to 5 (unable to/extreme problems). For each EQ-5D-5L, a single index value based on the validated French-specific value set (calculated between −0.5, worst health statement, to 1, best health statement) was derived [2]. In addition, patients were asked to indicate thanks to the EQ Visual Analogic Scale (VAS) their overall health on the day of questionnaire completion (from 0 = the worst health patient could imagine to 100 = the best health patient can imagine) (Additional file 2: EQ Visual Analogic Scale). The Beck Depression Inventory second version (BDI-II) was used to assess depression [3]. The scores vary between 0 and 63, with the following cut-off: 0–9: minimal or no depression, 10–18: mild depression, 19–29: moderate depression, 30–63: severe depression. Other data recorded were the usuals demographics data (age, gender), the disease phenotype and duration, and the treatments of WD.

The statistical methods are detailed in Additional file 3.

The present study was approved by the Institutional Review Board (IRB00003888, IORG0003254, FWA00005831) for the French Institute for Medical Research and Health (INSERM) (N°19-550).

## Results

Two hundred and fifty-seven patients were included in this study (Table 1). Patients had a mean age of 39.3 ± 12.6 years and were mainly men (n = 137, 53.3%). The mean disease duration was 19.6 ± 12.2 years. One hundred and fifty-three patient (59.5%) had a hepatic phenotype, 101 (39.3%) a hepatoneurologic (HN) one and only 3 (1.2%) had a presymptomatic disease. Patients were treated with Trientine 4HCL (n = 113, 44.0%), zinc sulfate (n = 73, 28.4%), D-Penicillamine (n = 51, 19.8%) and Trientine 2HCL (n = 5, 1.9%). Fourteen patients (5.4%) underwent a liver transplantation (LT). One presymptomatic patient (0.4%) has no treatment except a low copper diet.

**Table 1:** Patients’ characteristics

Overall (N = 257)	
**Sex**	
Female	120 (46.7%)
Male	137 (53.3%)
**Age at evaluation**	
Mean (SD)	39.3 (12.6)
Median (Q1, Q3)	38.0 (29.3, 48.4)
Min–Max	17.9–73.0
**Disease duration**	
Mean (SD)	19.6 (12.2)
Median (Q1, Q3)	18.8 (9.7, 28.1)
Min–Max	0.3–61.4
**Current phenotype**	
Hepatic	153 (59.5%)
Hepatoneurologic	101 (39.3%)
Presymptomatic	3 (1.2%)
**Treatment**	
TETA4HCL	113 (44.0%)
TETA2HCL	5 (1.9%)
DP	51 (19.8%)
Zinc salt	73 (28.4%)
LT	14 (5.4%)
No treatment	1 (0.4%)
**BDI**	
Mean (SD)	7.4 (9.4)
Median (Q1, Q3)	4.0 (0.0, 11.0)
Min–Max	0.0–56.0
**EQ5D5L index value**	
Mean (SD)	0.8 (0.3)
Median (Q1, Q3)	0.9 (0.7, 1.0)
Min–Max	−0.4–1.0
Missing	2
**EQ VAS (%)**	
Mean (SD)	75.1 (20.7)
Median (Q1, Q3)	80.0 (63.8, 90.0)
Min–Max	5.0–100.0
Missing	13

**Fig. 1:**
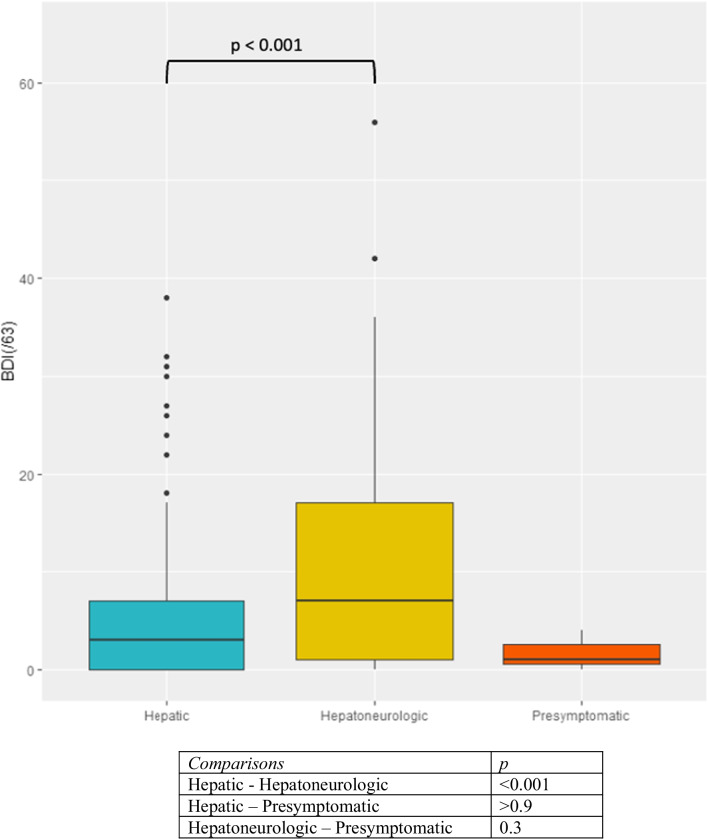
Boxplots representation of the distribution of BDI by clinical form of the disease and pairwise group comparison. Groups have been compared thanks to a Dunn post-hoc test with Bonferroni correction. BDI: Beck Depression Inventory

The mean EQ-5D-5L index value was 0.8 ± 0.3 and the mean EQ-VAS was 75.1% ± 20.7. The mean BDI score was 7.4 ± 9.4. Sixty-eight patients had no depression (27.5%), 160 (62.3%) had a minor to mild depression and 29 (11.3%) a moderate to severe depression. The BDI score was significantly higher in women (*p* = 0.01) and in HN patients (*p* < 0.001) (Fig. 1). No significant correlation was found with age (*p* = 0.2) or disease duration (*p* = 0.8).

**Fig. 2:**
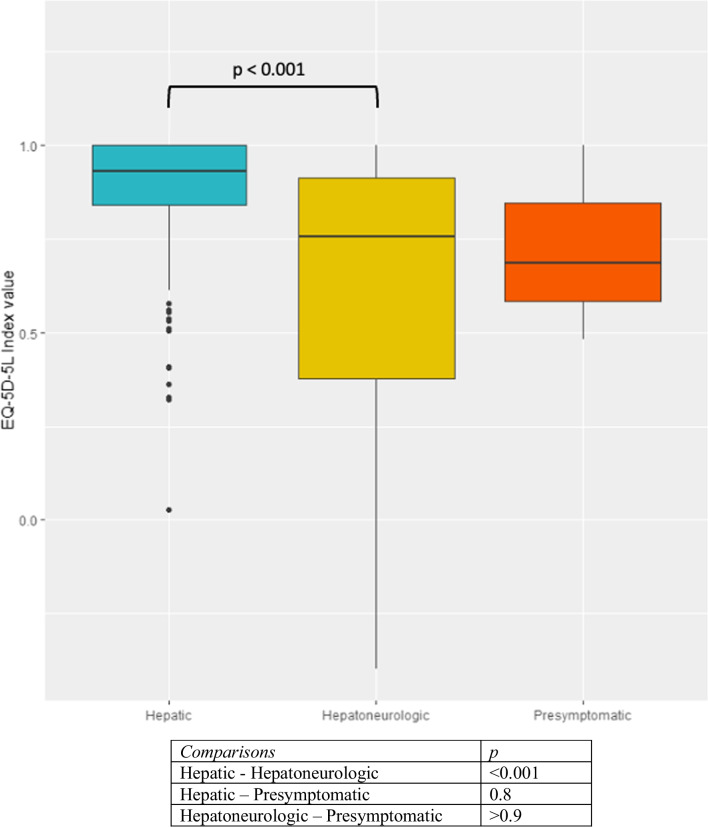
Boxplots representation of the distribution of EQ-5D-5L by clinical form of the disease and pairwise group comparison. Groups have been compared thanks to a Dunn post-hoc test with Bonferroni correction

**Fig. 3:**
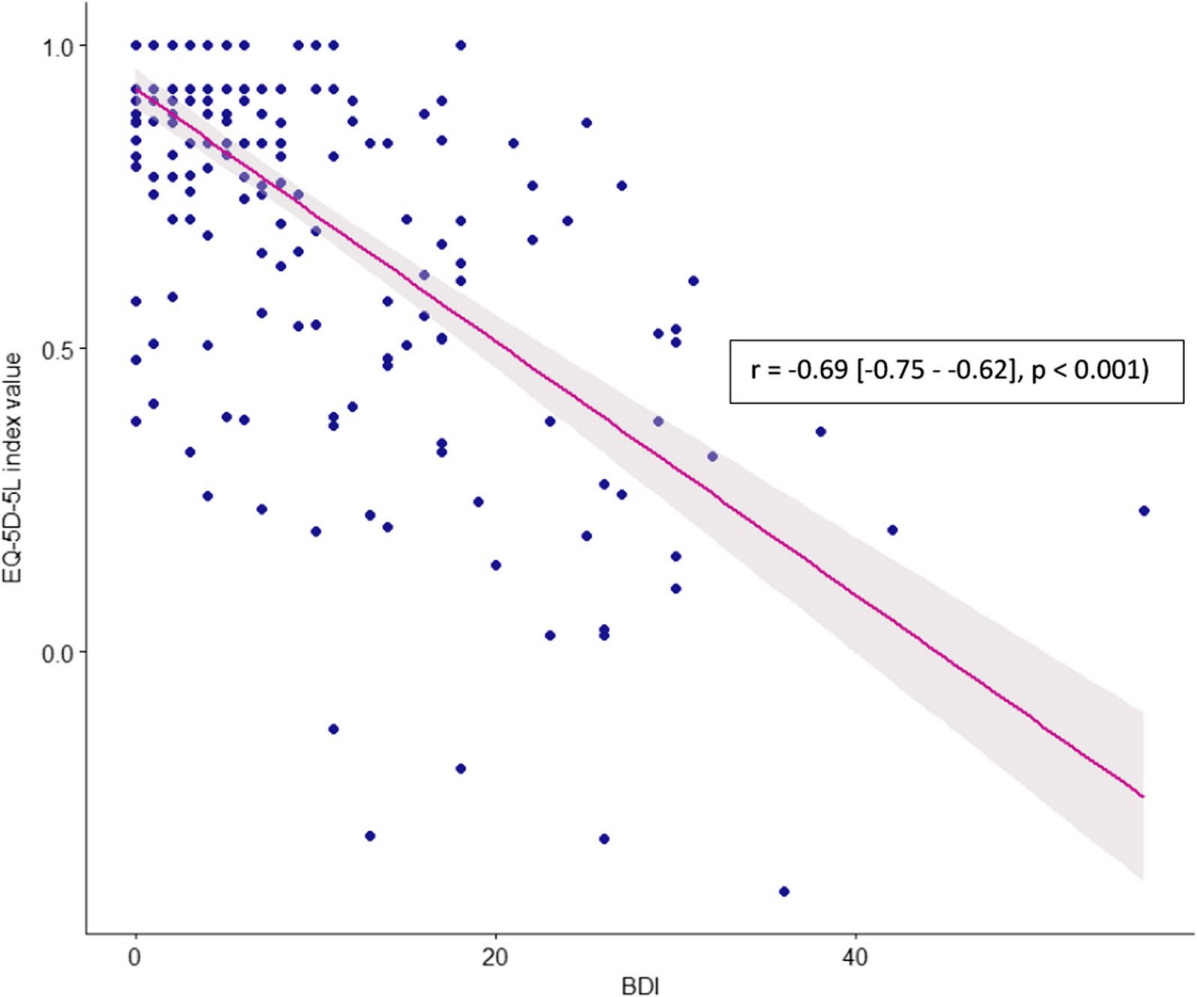
Correlation between EQ-5D-5L and BDI. Spearman correlation was used

The EQ-5D-5L index value was significantly worse in women (*p* = 0.03), in patients with a HN form of WD (*p* < 0.001) (Fig. 2) and in LT patients (*p* = 0.005). The EQ VAS was lower in the HN group of patients (*p* < 0.001). In multivariate analysis, having a HN form of WD significantly decreases the EQ-5D-5L value index score (−0.2 [−0.2 to −0.1], *p* < 0.001) and having an increase of one unit of BDI score also decreases the EQ-5D-5L value index score (−0.02 [−0.02 to −0.01], *p* < 0.001) adjusted to gender and age at evaluation (Table 2). The EQ-5D-5L value index was negatively correlated with the BDI score (r = −0.69 [−0.75 to −0.62], *p* < 0.001) (Fig. 3).

**Table 2:** Factors associated with poorest EQ-5D-5L index value

	Overall = 255 n (%) or mean (± SD)	Estimate (95% CI)	*p* value	Adjusted estimate (95% CI)	*p* value
Gender [Male]	137 (53.3%)	0.07 (0.002 to 0.1)	0.04	0.02 (−0.03 to 0.07)	0.5
Age at evaluation (years)	39.3 ± 12.6	−0.003 (−0.006 to −0.00002)	0.04	−0.001 (−0.003 to 0.0007)	0.2
Disease duration (years)	19.6 ± 12.2	−0.001 (−0.004 to 0.002)	0.4		
Hepatoneurological form	101 (39.3%)	−0.3 (−0.3 to −0.2)	< 0.001	−0.2 (−0.2 to −0.1)	**< 0.001**
Presymptomatic form	3 (1.2%)	−0.2 (−0.5 to 0.1)	0.3	−0.2 (−0.4 to 0.007)	0.06
Total BDI (/63)	7.4 ± 9.4	−0.02 (−0.02 to −0.02)	< 0.001	−0.02 (−0.02 to −0.02)	**< 0.001**

Bold value correspond to a statistically significant result

The following variables were included in the multivariate analysis: gender, age at evaluation, hepatoneurological form, presymptomatic form and total BDI

*BDI* Beck Depression Inventory

## Discussion

This large prospective cross-sectional study examined the QoL in WD patients followed in a national reference center. Our cohort included patients of different age over 18, gender, and diseases phenotype covering the entire spectrum of WD. Moreover, this single-center recruitment allowed a homogeneous medical care between patients in term of treatments, follow-up and accessibility to various aid such as psychological support, social worker, or physiotherapist. In this cohort, QoL of WD patients seems to be globally good. For comparison, the mean EQ-5D-5L index value was 0.84 in Belgian general population [4] and 0.897 in the Spanish one [5]. Interestingly, the mean VAS score in French general population was 77 ± 20.8 which is very close to our population (75.06 ± 20.68) [6]. In a cohort of Spanish patients with a chronic disease (diabetes) the mean EQ-5D-5L index value was 0.67 and the mean VAS score was 61.1 which is lower than in our cohort [7]. In our cohort, the length of follow-up and an easy access for the patients to an expert medical, paramedical and social care in a dedicated center to their rare disease, could explain the good QoL.

The QoL of our patients was related to the depressive state (higher in women and in HN patients) and, independently, to the HN phenotype of the disease. A recent systematic review of literature has showed that WD patients have a worse QoL than general population especially in patients with neurological form [8]. Nevertheless, this review included very heterogenous studies without differentiation of the disease phenotypes, or with a focus on specific treatments (some studies included only WD patients without treatment, some with medical treatment only, and one studied WD after LT) or with different scales for assessing the QoL. Concerning LT, we found that it could impact the QoL of patients but it concerned only 5.4% of the global cohort. Except for a paper on 24 WD patients that found no significant difference between WD patients who had a LT and the general population [9], no recent study have assessed the QoL in liver transplanted WD patients.

Schaefer et al. have studied retrospectively the health-related quality of life and the risk for depression in 68 WD patients [10]. Based on the PHQ-9 questionnaire, more than half of their patients were at risk for depression or suffered from depression and 21% were at high risk for major depressive disorder. These data contrast with our study; we found only 11% of patients with mild or severe depression. These differences could be explained by the larger size of our cohort (257 patients) and the design of the study: retrospective in Schaefer et al. and prospective in our, capturing more precisely the state of mind of the patients. Moreover, our center provides an easy access to psychologist care that could decrease the depression symptoms. Nevertheless, the QoL of their patient was similar to general population.

Different factors were associated with poorest QoL in the literature: female gender [10], depression [8, 11, 12], neurological form of WD [8, 10–13], treatments [10]. We did not found effect of the treatment in our cohort, and it can be explained by the homogenous management of our patients due to our monocentric recruitment. The influence of gender has also been found in our study, with a statistically significant lower EQ-5D-5L index value score in women in univariate analysis. Nevertheless, this difference disappears in the multivariate analysis. This could be explained by a link between depression assessed by the BDI score and the female gender like in our study. This “female preponderance” in the level of self-report depressive symptoms have been demonstrated in the general population in a meta-analysis [14]. On the other hand, the correlation between depression and impaired QoL in WD have been well demonstrated in a prospective study about 62 adults WD patients (*p* = 0.0017) [13]. Moreover, depression influence the QoL in different neurodegenerative disorders, even in the absence of significant motor and functional disability, enhancing its role as independent factor of QoL impairment like in our multivariate analysis [12]. The impact of the neurological phenotype was also found statistically significant in our study. There might be several explanations for this finding. Patients with neurological symptoms develop symptoms later, have a longer time between beginning of symptoms and diagnosis and are diagnosed later in life exposing them to the possibility of irreparable tissue damage which impact the QoL [10].

## Conclusion

At our knowledge, it is the largest study about QoL in WD. The prospective inclusion, the high number of patients for a rare disease, the use of validated score, the monocentric recruitment and the multivariate analysis provided the most robust data about QoL in WD. The main result was that QoL in WD was not very different with general population according to VAS score. As the HN form was significantly linked to a worse QoL and a higher rate of depression, neurological patients should be closely monitored to prevent and treat symptoms of depression that impact their QoL and proposed social and psychological support.

## Supplementary Information

**Additional file 1.** EQ-5D-5L questionary.

**Additional file 2.** EQ Visual Analogic Scale.

**Additional file 3.** Statistics.

## Data Availability

The datasets used and/or analyzed during the current study are available from the corresponding author on reasonable request.

## Abbreviations

BDI: Beck Depression Inventory
BDI-II: Beck Depression Inventory second version
HN: Hepatoneurologic
LT: Liver transplantation
QoL: Quality of life
VAS: Visual Analogic Scale
WD: Wilson’s disease
