# Supplementary material for: Quality of life and depression in Wilson’s disease: a large prospective cross-sectional study
Source: Orphanet J Rare Dis. 2023 Jun 29;18:168. doi: 10.1186/s13023-023-02777-4 (PMC10308610; doi:10.1186/s13023-023-02777-4)
Supplement: Supplementary file 1 — Additional file 1. EQ-5D-5L questionary. [file 13023_2023_2777_MOESM1_ESM.docx]

**Supplementary data 1 – EQ-5D-5L questionary**

| ***Under each heading, please tick the ONE box that best describes your health TODAY.*** | |
| --- | --- |
| ***MOBILITY*** |  |
| *I have no problems in walking about* | *θ* |
| *I have slight problems in walking about* | *θ* |
| *I have moderate problems in walking about* | *θ* |
| *I have severe problems in walking about* | *θ* |
| *I am unable to walk about* | *θ* |
| ***SELF-CARE*** |  |
| *I have no problems washing or dressing myself* | *θ* |
| *I have slight problems washing or dressing myself* | *θ* |
| *I have moderate problems washing or dressing myself* | *θ* |
| *I have severe problems washing or dressing myself* | *θ* |
| *I am unable to wash or dress myself* | *θ* |
| ***USUAL ACTIVITIES (e.g. work, study, housework, family or leisure activities)*** |  |
| *I have no problems doing my usual activities* | *θ* |
| *I have slight problems doing my usual activities* | *θ* |
| *I have moderate problems doing my usual activities* | *θ* |
| *I have severe problems doing my usual activities* | *θ* |
| *I am unable to do my usual activities* | *θ* |
| ***PAIN / DISCOMFORT*** |  |
| *I have no pain or discomfort* | *θ* |
| *I have slight pain or discomfort* | *θ* |
| *I have moderate pain or discomfort* | *θ* |
| *I have severe pain or discomfort* | *θ* |
| *I have extreme pain or discomfort* | *θ* |
| ***ANXIETY / DEPRESSION*** |  |
| *I am not anxious or depressed* | *θ* |
| *I am slightly anxious or depressed* | *θ* |
| *I am moderately anxious or depressed* | *θ* |
| *I am severely anxious or depressed* | *θ* |
| *I am extremely anxious or depressed* | *θ* |
