# Supplementary material for: Quality of life and depression in Wilson’s disease: a large prospective cross-sectional study
Source: Orphanet J Rare Dis. 2023 Jun 29;18:168. doi: 10.1186/s13023-023-02777-4 (PMC10308610; doi:10.1186/s13023-023-02777-4)

**Supplementary data 2 - EQ Visual Analogic Scale**

- **We would like to know how good or bad your health is TODAY.**
- **This scale is numbered from 0 to 100.**
- **100 means the best health you can imagine. 0 means the worst health you can imagine.**
- **Please mark an X on the scale to indicate how your health is TODAY.**
- **Now, write the number you marked on the scale in the box below.**


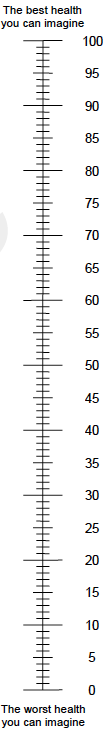

Supplement: Supplementary file 2 — Additional file 2. EQ Visual Analogic Scale. [file 13023_2023_2777_MOESM2_ESM.docx]
