# Supplementary material for: Quality of life and depression in Wilson’s disease: a large prospective cross-sectional study
Source: Orphanet J Rare Dis. 2023 Jun 29;18:168. doi: 10.1186/s13023-023-02777-4 (PMC10308610; doi:10.1186/s13023-023-02777-4)
Supplement: Supplementary file 3 — Additional file 3. Statistics. [file 13023_2023_2777_MOESM3_ESM.docx]

**Supplementary data 3 – Statistics**

Qualitative data were described as frequencies and percentages, and quantitative data using the mean, standard deviation, median, range and interquartile range. Correlations of two quantitative variables were estimated using Spearman’s correlation with 95% confidence intervals. Comparisons of a quantitative variable between two groups were performed with Wilcoxon-Mann-Whitney’s test and between more than two groups with Kruskal-Wallis’ test. Two by two comparisons for these three variables between phenotypes were performed using post-hoc Dunn’s test with Bonferroni correction. Univariate and multivariate linear regression were realized to identify factors associated with EQ5D5L index value. Covariates selected in the multivariate linear regression were already known to be linked to the dependent variable or were significant at a threshold of p<0.2 in the univariate linear regression. All tests were bilateral and performed with a two-sided significance level of p<0.05. All statistical analyses were performed using R version 4.0.3.
